# Bibliographical

**Published:** 1877-03

**Authors:** 


### BIBLIOGRAPHICAL.

We have just received from the publishers a copy of "A Treatise on Operative Dentistry," by J. Taft, third edition, 128 illustrations.

The publishers have done their work well. In paper, type, binding, etc., they have made a very neat volume, and on this account, if no other, every dentist should have a copy in his library.

If it will in any wise aid the student or practitioner in making professional advancement, it will contribute to the gratification of the author.

